# Cannabis Use, Cannabis Use Disorder, and Comorbid Psychiatric Illness: A Narrative Review

**DOI:** 10.3390/jcm10010015

**Published:** 2020-12-23

**Authors:** Deborah Hasin, Claire Walsh

**Affiliations:** 1 New York State Psychiatric Institute, New York, NY 10032, USA; claire.walsh@nyspi.columbia.edu; 2 Department of Epidemiology, Mailman School of Public Health, Columbia University Medical Center, New York, NY 10032, USA

**Keywords:** cannabis, comorbidity, cannabis use disorder, co-occurring disorder

## Abstract

Background: The landscape of attitudes, legal status and patterns of use of cannabis is rapidly changing in the United States and elsewhere. Therefore, the primary aim of this narrative review is to provide a concise overview of the literature on the comorbidity of cannabis use and cannabis use disorder (CUD) with other substance use and psychiatric disorders, and to use this information to accurately guide future directions for the field. Methods: A literature review of PubMed was conducted for studies relating to cannabis use, CUD, and a co-occurring psychiatric disorder. To provide an overview of representative data, the literature review focused on national-level, population-based work from the National Epidemiologic Survey on Alcohol and Related Conditions (NESARC) and National Survey on Drug Use and Health (NSDUH) surveys. Considering rapidly changing cannabis laws, recent (past five-year) studies were addressed. Results: A strong body of literature shows associations between cannabis use and CUD with other drug use, psychosis, mood disorders, anxiety disorders, and personality disorders. The strongest evidence of a potential causal relationship exists between cannabis use and psychotic disorders. While some evidence shows potential directionality between cannabis use and mood and anxiety disorders, results are inconsistent. Studies have established higher rates of CUD among those with personality disorders, but little about the specifics of this relationship is understood. Conclusions: Although the general population in the United States increasingly perceives cannabis to be a harmless substance, empirical evidence shows that cannabis use is associated both with CUD and comorbid psychiatric illness. However, there is mixed evidence regarding the role of cannabis in the etiology, course, and prognosis of a co-occurring disorder across all categories of psychiatric disorders. Future research should expand on the existing body of literature with representative, longitudinal data, in order to better understand the acute and long-term effects of cannabis on comorbid psychiatric illness.

## 1. Introduction

Cannabis is one of the most widely used psychoactive substances in the United States (U.S.), with around 43.5 million people over the age of 12 reporting past-year use and around 124 million people reporting lifetime use in 2018 [1,2]. The legal status of cannabis in the U.S. is rapidly changing, with a total of 33 U.S. states permitting adult use of medical cannabis and 11 states additionally permitting adult recreational use in 2020 [3]. Globally, an estimated 188 million people used cannabis within the past year in 2017, with trend rates in use rising substantially in the Americas and Asia [4]. Although trends in cannabis use have not increased at the same rate in Europe, cannabis remains the most commonly used illicit drug there [4]. For example, cannabis accounted for 71% of all illegal drug seizures in England and Wales in the fiscal year 2018–2019 [5]. Furthermore, the changing legalities in the United States has stimulated debate in Europe regarding the advantages and disadvantages of medical and recreational cannabis [6]. No country in the European Union currently permits cannabis for medical or recreational use. However, cannabis use is decriminalized in countries such as Portugal and the Netherlands [7]. Data show that youth across Europe perceive cannabis use as risky, but this perception may be moderated by peer use [8]. Despite an increasing perception among the U.S. public of cannabis as a safe substance [9], both adverse mental and physiological effects of cannabis use can occur [10,11,12,13]. In the U.S. general population, the prevalence of medical and recreational cannabis use, as well as cannabis use disorder (CUD), is increasing [14]. Levels of delta-9-tetrahydrocannabinol (THC) concentration in cannabis products are also increasing in the U.S. [15], and in Europe [16,17,18]. Furthermore, while a commonly-held assumption is that few cannabis users will develop cannabis use disorder [19], CUD now occurs in 20–30% of users [20,21,22].

Studies dating back to the 1980s show a high degree of comorbidity of substance and psychiatric problems among treated patients [23]. These findings were originally assumed to be due to Berkson’s bias, i.e., that those with multiple conditions more likely to enter treatment than those with only a single condition of primary interest [24]. However, the first large-scale general population study of specific substance and psychiatric disorders in the U.S., the Epidemiologic Catchment Area (ECA) 5-site study [25], indicated that psychiatric and substance use disorders (SUDs) were also highly comorbid in adults in the general population [24,25]. Additional findings in several more recent nationally representative surveys have confirmed the association of psychiatric and substance use disorders, and expanded on the specificity of the associations [26,27,28,29,30,31,32,33,34,35]. In these general population studies, comorbidity was defined as evidencing both types of disorder within the past year, or on a lifetime basis. Understanding the comorbidity of substance and psychiatric disorders is important to guide clinicians, inform the delivery of treatment services and suggest etiological factors. With the changing legal landscape and increasing prevalence of cannabis use, examining the comorbidity of psychiatric disorders with cannabis use has become especially important. 

Psychiatric and substance use disorders are each associated with disability and impaired functioning [27,30,31,36,37,38]. For example, those with depression and those with an SUD have been shown to score significantly below the population-based mean on the 12-Item Short-Form Health Survey version 2 (SF-12v2) [30,31], a reliable measure of social and emotional functioning, as well as disability. Thus, individuals with comorbid substance and psychiatric disorders may be at risk of greater disability and more greatly impaired functioning than those with single disorders [36,39]. Because of this, a closer examination of the current evidence regarding the association of CUD with other psychiatric illnesses is warranted.

Although strong evidence has suggested that cannabis use and CUD are associated with psychiatric comorbidities [28], the complexities of this association are not fully understood, and even inconsistent at times. Specifically, national data have consistently shown that those with past-year and lifetime CUD are at an elevated risk of other illicit substance use, psychosis, mood disorders, anxiety disorders, and personality disorders, in comparison to individuals with no CUD [28,40,41,42,43,44]. However, the nature of the relationship between cannabis use, CUD and other psychiatric disorders remains unclear, with empirical findings on the etiology and course of CUD with a co-occurring disorder producing mixed findings. Therefore, this narrative review provides an examination of the current literature on cannabis use, CUD, and dual diagnoses, in order to summarize what is known from large scale, population-based studies, and establish future directions to guide research, practice and policy. The objectives of the review are to (1) provide an overview of information on the association of cannabis use and CUD with other psychiatric disorders, and (2) concisely identify particular risk factors related to the course of the disorders when comorbid, and (3) summarize the related unclear or inconsistent results. This material is organized by the category of psychiatric diagnosis.

## 2. Methods

**Figure 1:**
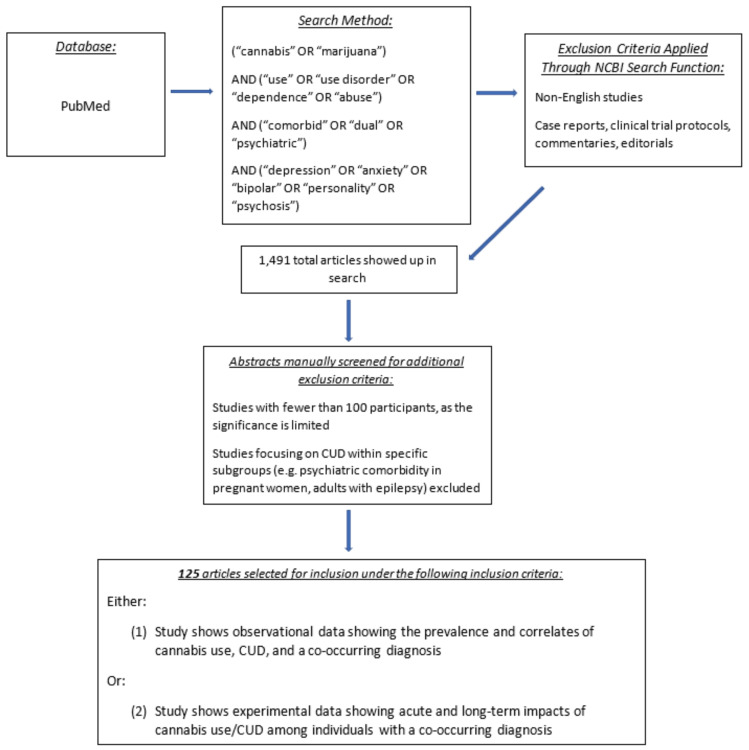
PubMed search strategy to identify studies or meta-analyses with data on cannabis use, cannabis use disorder (CUD), and a co-occurring disorder.

A search of PubMed was conducted for studies pertaining to cannabis use, CUD, and co-occurring disorder using defined terms. PubMed was selected as the primary article database, since it is currently one of the largest collections of peer-reviewed, biomedical research, containing over 30 million sources as of writing [45]. A similar initial search was also conducted in the Google Scholar database. Upon screening of the first 100 search results, this database produced similar articles. Searches included the phrases “cannabis use”, “cannabis use disorder”, in combination with “comorbidity”, “dual diagnosis”, “psychiatric”, and comorbid diagnoses of interest: “depression”, “anxiety”, “bipolar”, “personality”, “psychosis”, “substance”. Studies with samples smaller than 100 participants were excluded, as were studies of highly specific samples or subgroups (i.e., studies with a sample only consisting of individuals with a physical condition such as heart failure, or specific study settings such as group homes). While addressing cannabis use and comorbid psychopathologies within these specific groups is important, the primary aim of the review is to provide an overview of large, nationally representative data to remain generalizable. Additional exclusion criteria included non-English studies, commentaries, clinical trial protocol lists, case reports, and opinion pieces. These exclusion criteria and search terms were applied through the “My NCBI Filters” feature on PubMed. Selected search terms were required to be in the title, abstract, and/or key words of the articles. The non-empirical article types described above were automatically left out of the search through the computerized filter function. Although no formal range of dates was applied, studies published within the past five years were the focal point of the review, in light of rapidly changing cannabis policies. Older studies were incorporated as necessary to provide additional information or context. The exact search text used in PubMed is detailed in Figure 1. 

The search was conducted on 11 August 2020 and drew 1491 total results. Articles were sorted by recency of publication to prioritize the most relevant literature. Of the 1491 articles, the screening of article titles and abstracts was conducted by CW to determine eligibility for inclusion, e.g., studies that included fewer than 100 participants were excluded. Furthermore, in order to summarize relevant, nationally representative findings for the purpose of generalizability, articles that focused on cannabis use and a co-occurring disorder within specific subgroups (e.g., CUD and psychiatric comorbidity in pregnant women, adults with epilepsy, or those with chronic inflammation) were excluded. To organize the findings, relevant articles on cannabis use and comorbidity were added to a list on an Excel spreadsheet, and sorted by related dual diagnoses. After excluding articles on the basis described above, 125 articles were selected for inclusion. 

After the PubMed search was conducted and relevant articles were incorporated, additional papers were retrieved to provide more specific information pertaining to specific points regarding cannabis policy, comorbid illnesses, and etiology of those illnesses.

Since many of the included studies used data from two major nationally representative US survey series, the surveys are described briefly here. One series consisted of the National Epidemiologic Survey on Alcohol and Related Conditions (NESARC) surveys [28,40]. These surveys included large, nationally representative samples of household residents who were assessed for patterns of lifetime and current alcohol use, illicit drug use, alcohol use disorders, drug use disorders, and many psychiatric disorders [46,47]. The surveys provide detailed data on substance use and psychiatric disorders. The 2001–2002 NESARC included 43,093 adults. Approximately a decade later, a survey was conducted with a similar sample design and measures, the NESARC-III, which included 36,309 adults. 

Another important source of information on substance use and comorbidities is the series of National Survey on Drug Use and Health (NSDUH). These yearly surveys, sponsored by the Substance Abuse and Mental Health Services Administration (SAMHSA), have provided national prevalences of substance use and related psychiatric and health issues since 1971 [48], and are therefore an important source for identifying comorbidity trends at the national level. The NSDUH surveys include considerable detail on drug use patterns, drug use disorders, and some information on alcohol use and alcohol use disorders. Some psychiatric conditions are also covered in NSDUH, although fewer and in less detail than the NESARC surveys. NSDUH surveys are often used to examine trends over time, in particular patterns of drug use and potentially related variables. Moreover, nationally representative trends data allows for identifying groups at an elevated risk of experiencing a comorbid psychiatric illness, for example youth [49,50], people in poverty [51], and non-medical opioid users [52]. 

## 3. Results

### 3.1. The Association of CUD with Other SUDs

CUD is highly comorbid with other SUDs. NESARC results (Table 1) show that around half of those with past-year CUD also met diagnostic criteria for an alcohol use disorder (OR 7.8) or nicotine dependence (OR 5.1) [40]. NESARC-III findings (Table 1) also show strong associations between past-year CUD and other SUD (OR 6.0–9.3) [28]. 

Later NESARC-III analyses have illustrated extensive polysubstance involvement, showing that DSM-5 CUD is associated with higher prevalences of other substance use disorders across all drug classes [42]. Notably, past-year CUD was associated with an elevated risk of a co-occurring cocaine (aOR 9.3), sedative (aOR 5.1), stimulant (aOR 4.3), club drug (aOR 16.1), and opioid (aOR 4.6) use disorders [42]. However, CUD and a concurrent heroin or other drug use disorder were not significantly associated [42]. In addition, age at onset of cannabis use was two years earlier, on average, among those with CUD compared to those without CUD (15.7 years old and 17.7 years old, respectively) [42]. Finally, cannabis was used prior to any other substance class regardless of CUD status [42]. 

Although there is less information on CUD and rates of other specific SUDs in the NSDUH, trend data allow for identification of potential patterns of polysubstance use. NSDUH data show that when cannabis is used for the first time prior to alcohol or cigarettes, youth are more likely to later show heavy patterns of cannabis use and develop CUD. [53]. Consistent with NESARC, NSDUH data show strong associations between CUD symptoms and concurrent nicotine dependence, and that concurrent cannabis and cigarette use is associated with a greater number of CUD symptoms compared to non-cigarette smokers [54]. Furthermore, both cannabis users and those with CUD are significantly less likely to quit smoking cigarettes than non-users [55]. Data also show that in adolescents, concurrent use of cannabis and tobacco is more common (5.4%) than use of either cannabis or tobacco only (2.2% and 3.9%, respectively) [56]. However, data for those over the age of 18 show that tobacco use only is more prevalent (24.0%) than co-use (5.2%) or use of cannabis only (2.3%). Analysis of adult data also shows that co-use of cannabis and tobacco is increasing over time [57], although the adolescent trend data do not report a significant increase in co-use over time [56]. Future studies of NSDUH data should identify cannabis use, CUD, and potential co-use patterns of cocaine, sedatives, stimulants, club drugs, opioids, heroin, and ‘other’ drugs. The causal role of cannabis as a “gateway drug” to other illicit substance use is unclear. Some studies show the onset of cannabis use prior to other substances [42,58,59]. For example, a 25-year longitudinal study found a strong association between adolescent use of cannabis and later other drug use, and that the odds of later other illicit drug use increased as cannabis was used more frequently (at least weekly versus at least monthly or less than monthly) [60]. 

Because both medical cannabis and prescribed opioids are now legal for patients experiencing chronic pain in many locations, understanding how these two substances are used concurrently is a high priority. The comorbidity of CUD and opioid use disorder (OUD) has been shown in national data, indicating that cannabis use is associated with greater non-medical opioid use in pain patients (aOR 2.99) [61], and in the general population [52]. This is consistent with some findings in veteran samples, revealing that a CUD diagnosis is strongly associated with greater opioid prescription fills [62], although another study has shown the opposite: that co-occurring CUD and OUD are associated with less prescription fills than in veteran patients with OUD alone [63]. Also, recent data analyzing daily self-report of drug use among problematic substance users show that regardless of pain level, on days where non-medical opioids were used, the odds that cannabis was used on the same day were around double (aOR 1.86) [64]. 

Moreover, cannabis remains the most commonly used drug among those who drink alcohol [2]. Therefore, studies determining how changes in the legal status in cannabis will impact the simultaneous use of alcohol are needed, as well as the frequency and severity of alcohol use disorder (AUD) and CUD. The presence of any CUD significantly increases odds of a co-occurring AUD [28,40], and over half of those with a past-year CUD have a comorbid AUD [40], suggesting the risk of simultaneous use. Numerous studies report the simultaneous use of alcohol and cannabis among adolescents [65,66,67]. Data from the National Alcohol Survey (NAS) show that simultaneous use of cannabis and alcohol is associated with greater quantities of alcohol use, elevated risk of drunk driving, alcohol-related social problems, and harm to self [68]. Further studies have identified potential risks of co-use of alcohol and cannabis. One review identified that individuals who co-use alcohol and cannabis experience more alcohol-related problems than those who use alcohol alone [69], and similarly reported findings regarding elevated risk of drunk driving when both alcohol and cannabis are used [69]. Because of these risks, and given that the presence of more than one SUD is associated with poorer prognosis than one SUD alone [70], additional clinical research is necessary to identify effective intervention strategies for individuals experiencing problematic co-use of alcohol and cannabis. 

### 3.2. CUD and Psychotic Disorders

Psychotic disorders are rare in the general population [71,72] and their lifetime prevalence varies somewhat across studies. However, the substantial burden of psychotic disorders on the individuals afflicted, their caretakers, and economic costs to society as a whole is clear [72]. While the nature of the relationship between cannabis use and psychosis has been debated, reviews and meta-analyses indicate that cannabis use may be one of the causal factors in the risk for incidence and poor prognosis of psychosis [16,73]. Different lines of evidence suggest that the relationship may be causal, including time order, dose–response relationship, and studies ruling out potential confounders. Cannabis use is associated with treated psychotic disorders [73,74,75,76,77,78,79,80]. THC is the component that increases risk [79,81,82,83,84,85,86]. Most of these studies addressed cannabis use rather than cannabis use disorders. For example, a 6-country study showed strong associations of cannabis frequency and THC potency with first-episode psychosis (OR 4.8) [75]. 

Reviews and meta-analyses of prospective studies show that previous cannabis use predicts treated first-episode psychosis [73,74,79,87]. Studies addressing potential reverse causation either ruled it out [88,89,90] or found bi-directionality, i.e., partial causality [91,92]. In addition, among patients with psychotic disorders, cannabis is among the most widely abused substances [93,94,95]. While in general, substance use disorders predict psychosis relapse [96], meta-analyses specifically focused on cannabis show that continued cannabis use among patients with psychotic disorders predicts psychotic symptom severity, worse functioning and greater risk of relapse (defined as hospitalization) [80,97]. This may be due to the potential for cannabis to directly exacerbate psychosis symptoms [80] or to adverse effects on antipsychotic medication adherence [97,98]. 

Numerous studies have addressed a dose–response relationship between cannabis and psychosis in terms of frequency of use, THC potency, or both. A systematic review (2007) [99] and a 2016 meta-analysis [100] found that greater frequency of cannabis use was associated with greater risk for psychosis. The odds of psychosis were significantly greater among those using high-potency cannabis compared to low-potency users [101]. We re-computed the ORs in this paper [101] within frequency strata, finding that ORs for the risk for psychosis by THC potency remained strong and significant within all frequency levels (OR = 3.9–4.7). Moreover, high- but not low-potency cannabis use predicts poor antipsychotic medication adherence [102]. Additional reports also indicate increased risk for psychosis from products with higher THC potency. One such study utilizing Danish health records linked an increase in cannabis-induced psychosis since 2006 to an increase in both frequency of use and THC concentration over time [77]. A recent study using NESARC and NESARC-III data showed that participants reporting that a doctor or other health professional told them they had schizophrenia or a psychotic episode were more likely to be frequent cannabis users and have a current CUD diagnosis than other participants [103].

On the other hand, meta-analyses have shown that cannabis use is associated with better cognitive function in patients with schizophrenia, for example, one meta-analysis that found that patients with schizophrenia who reported a history of using cannabis performed better on visual and working memory tasks than those who did not [104]. However, these data were limited and the findings have not always been replicated with larger pooled samples [105]. Additionally, much of the literature focuses on the relationship of cannabis use to psychosis. Data remain limited on the role and severity of CUD in relation to psychotic disorders. Thus, studies are needed in order to better understand the role of cannabis and CUD in the incidence, course and cognition of psychotic disorders.

Many issues remain unresolved about the relationship between cannabis use and psychotic disorders. Because of the serious, chronic and impairing nature of psychotic disorders such as schizophrenia and schizo-affective disorders, research on mechanisms of the effect of cannabis on psychosis and how that may differ across population subgroups is a highly important area of ongoing research. 

### 3.3. CUD and Mood Disorders

Numerous studies indicate a higher prevalence of mood disorders among those with a CUD compared to others in the general population [28,40,106,107,108,109]. Specifically, NESARC data show higher levels of major depressive disorder (MDD), bipolar I, bipolar II, and DSM-IV dysthymia in those with both a past-year and lifetime CUD, with strongest odds ratios (ORs) reported for major depression (OR 1.9 lifetime, 1.8 past-year) and bipolar I (OR 2.5 lifetime, 3.1 past-year) [40]. These findings are consistent with a later analysis using data from the NESARC-III, indicating associations between DSM-5 CUD and MDD, bipolar I, and bipolar II, with a stronger association for bipolar I than bipolar II (past-year OR: 5.0 vs. 2.7, lifetime OR: 3.8 vs. 2.8) [28]. 

Furthermore, a three-year follow-up study of NESARC participants found that a CUD diagnosis was associated with later severity of MDD. Specifically, both baseline cannabis use and CUD predicted a greater number of MDD symptoms three years later, compared to nonusers [110]. There was no significant difference between groups in overall MDD remission rate or quality of life at three-year follow-up, but cannabis users were more likely to experience some specific symptoms, including anhedonia, sleep problems, changes in body weight, and psychomotor agitation or retardation at follow up. These depressive symptoms have substantial overlap with symptoms of DSM-5 cannabis withdrawal syndrome (CWS) [111] (see Table 2), and are commonly reported symptoms among patients entering psychiatric or primary care. CWS is present in ~12% of frequent cannabis users [112], is associated with major depression, and can cause significant impairment [113,114]. Due to the overlap in symptoms, frequent cannabis users who are unaware of the existence of cannabis withdrawal could mistake its symptoms for those of depression, and continue using cannabis in an effort to self-medicate the symptoms, although prospective studies show that among psychiatric patients with depression, cannabis use predicts a worse course over time [115,116]. 

Analyses of NSDUH data also indicate associations between cannabis use and depressive episodes [44,49,50,109]. Using data from 728,691 participants from 2002–2012 in the National Survey on Drug Use and Health (NSDUH), both daily and non-daily cannabis use was more than twice as prevalent in those with past-year MDD compared to those without MDD (past 30-day use: 18.94% vs. 8.67% in 2017, *p* < 001) [117]. Additionally, participants with MDD perceive cannabis as less risky compared to those without, and while perception of risk decreased in those with MDD and those without MDD from 2005 to 2017, the decrease was significantly greater in those with MDD than in others (OR 0.90 vs. 0.93, *p* < 001) [117]. Finally, data from the National Health and Nutrition Examination Survey (NHANES) have shown that the strength of the association between cannabis use and depression has increased over time, and that participants with MDD were more likely to experience daily or near-daily cannabis use than those without [118], consistent with the prior NSDUH findings.

While causality cannot be inferred from these cross-sectional studies, twin modeling has evaluated whether CUD changes the risk of the development of MDD. One study analyzing 565 monozygotic twin pairs and 640 dizygotic twin pairs (total n = 2410) found that among the monozygotic pairs that included 1 twin with CUD and 1 twin without CUD, the twin with CUD was significantly more likely to have MDD (46.0%) than their co-twin without CUD (28.12%) [119]. These results are consistent with a meta-analysis of longitudinal data, finding cannabis users at an increased risk for developing depression [120] compared to non-users. However, whether these findings are due to causality or shared risk factors remains unclear. In addition, studies that can clarify contradictory evidence of the role of gender in cannabis use and the risk of developing depression [121,122] are also needed.

Three NESARC studies have evaluated the relationship of CUD and bipolar disorders [28,40,123]. All studies consistently showed associations between bipolar I and bipolar II with CUD, with comparatively stronger association of bipolar I with CUD (Table 1). However, these results may not be unique to cannabis use specifically, as prior research has shown higher prevalence of SUDs in people with bipolar I than any other psychiatric diagnosis [124,125]. Additional analyses have shown that individuals with bipolar disorder and a co-occurring CUD are at an elevated risk for having another concurrent SUD, as well as antisocial personality disorder, in comparison to those with bipolar disorder and no CUD [123]. A recent meta-analysis of 53 studies of bipolar disorder patients (51,756 pooled participants) found that around 20% of the samples qualified for lifetime CUD, higher than general population estimates [126]. Cumulatively, these findings provide strong evidence of the association of CUD with bipolar disorders. Additionally, current experimental data show that among individuals with bipolar I, the presence of an SUD was not a predictor of time to recovery from depression, although SUD presence was associated with a greater likelihood of switching from depression to a manic, mixed, or hypomanic state [127]. However, this study did not assess cannabis use or CUD specifically.

### 3.4. CUD and Anxiety Disorders

Anxiety disorders are the most prevalent mental illness in the United States, impacting over 30% of adults in their lifetime [128]. The prevalence of these disorders increases the importance of understanding their relationship to cannabis use, and CUD.

When taken in high doses, THC can cause symptoms of anxiety, as well as panic attacks [129,130], suggesting the potential to exacerbate anxiety disorders such as panic disorder and generalized anxiety. However, cannabidiol (CBD) has been shown to reduce symptoms of anxiety [131], showing a possible complementary effect [132]. Emerging evidence suggests cannabis as a treatment for anxiety disorders, particularly post-traumatic stress disorder (PTSD), specifically, one systematic review that identified therapeutic benefit of medical cannabis for PTSD symptoms, including internalizing symptoms and nightmares [133]. However, a recent longitudinal study among a veteran population with PTSD who also used non-medical opioids found that cannabis use had no significant impact on PTSD symptoms at follow-up [134]. Dose may play a role in the way cannabis impacts PTSD symptoms acutely and long-term, since at low doses, THC has been shown to reduce stress-induced corticosterone release and amygdala activity in the brain, thus aiding in PTSD stress symptoms [135]. However, this is complicated by potential adverse effects of long-term cannabis use, such as the potential for downregulation of cannabinoid 1 receptors [135]. Because this can impair the stress mechanisms in individuals with PTSD, this opens the potential for long-term negative impacts of cannabis use on PTSD symptoms. Nonetheless, interpretation of findings from prospective studies is complicated by the potential for long-term adverse effects such as the later development of CUD [136], highlighting the need for the monitoring of medical cannabis use among clinical populations. In particular, NESARC data have shown that those with PTSD are significantly more likely to develop CUD compared to those without (9.4% vs. 2.2%) [41].

There is some evidence from NESARC data that while social anxiety disorder and CUD may not be as strongly associated as other anxiety disorders, SAD may be a predictor of cannabis dependence [137]. This could be due to the use of cannabis as a coping mechanism in social situations [138]. Furthermore, SAD and a co-occurring CUD can lead to a poorer prognosis of SAD symptoms long term [137].

Both the NESARC and NESARC-III have established comorbidity of CUD and anxiety disorders, including panic disorder with agoraphobia, generalized anxiety disorder (GAD), and PTSD [28,40]. However, the directionality of effect between cannabis use, CUD, and anxiety disorders remains unclear. While one study based on retrospective information obtained in adulthood suggested that the onset of an anxiety disorder occurs prior to first onset of cannabis dependence symptoms [139], numerous other prospective studies have illustrated the risk of adolescent cannabis use on the later development of an anxiety disorder [140,141]. In particular, one of these studies involving adolescents found an association between daily use of cannabis in mid-teens, and the presence of an anxiety disorder at age 29 (aOR 2.5) [141]. However, in general, the role of cannabis in the etiology, prognosis, and treatment of anxiety disorders remains unclear, and additional research is needed to clarify these issues.

### 3.5. CUD and Personality Disorders

Individuals with personality disorders think and behave in a way that deviates from cultural expectations and that causes distress [111]. Personality disorders can impact an individual over an extended period of time, affecting concepts of identity, control of emotional responses, and relationships with other people [111]. DSM-5 identifies 10 personality disorders. NESARC data show associations between current (past-year) CUD and all personality disorders (OR 2.6–7.2), with strongest associations reported between CUD and dependent or antisocial personality disorder [40]. These associations between current CUD and a personality disorder have also been established in NESARC-III (OR 3.8–5.0), reporting stronger ORs of CUD and borderline or antisocial personality disorder in comparison to schizotypal personality disorder. Furthermore, a NESARC analysis of 5196 participants with a personality disorder found that 9% of the sample reported past-year cannabis use, with the highest proportion of cannabis users in those with a Cluster B personality disorder (antisocial; borderline; histrionic; narcissistic) compared to Cluster A (paranoid; schizoid; schizotypal), or Cluster C (avoidant; dependent; antisocial) [43]. These findings support an emerging line of evidence that personality disorder traits (i.e., interpersonal reactivity, an RDoC construct) could partially explain the variance in associations between specific personality disorders and CUD [142], and that cannabis may be used as a way to self-medicate. Cannabis use among those with a personality disorder was associated with an increased rate of other SUDs three years later [43]. However, there were no strong associations between cannabis use and other later psychiatric disorders, suggesting that cannabis users with a personality disorder may only be at significantly elevated risk for additional SUDs. Additional longitudinal research is lacking on the long-term outcomes of cannabis use and use disorder among those with a concurrent personality disorder.

Individuals with any personality disorder are significantly more likely to have a past-year CUD than those without (OR 3.8–5.0) [28]. NESARC-III data show that borderline personality disorder has the strongest association, compared to antisocial and schizotypal [28], and these findings are consistent with a twin study [143], as well as numerous other studies [144,145]. However, the specific mechanisms behind CUD and co-occurring personality disorders are yet to be understood. A twin study in the Norwegian general population found that genetics may play a role in cannabis use, cannabis use disorder, and some personality disorder traits, but not others [143]. Genetic and environmental correlations between personality disorder traits and cannabis use suggest that genetic risks in borderline and antisocial personality disorder traits accounted for significant variance in cannabis use, however not for schizoid or dependent personality disorder traits [143]. Conversely, schizoid and dependent traits were associated with lower levels of cannabis use [143]. Thus, much remains to be clarified about the relationship of cannabis use and cannabis use disorders to personality disorders.

## 4. Discussion

Due to the rapidly changing legal status of cannabis, the review aims to summarize the potential risks of frequent cannabis use by synthesizing the recent literature reporting on cannabis use, CUD, and comorbid psychopathologies. Although cannabis is increasingly perceived as a harmless substance, empirical evidence suggests considerable potential for adverse effects, including an increased risk for a host of concurrent psychiatric illnesses. Identifying these risks is more relevant now than ever, in light of the rapidly changing legal status of medicinal and recreational cannabis. While a large body of research ties cannabis use and CUD to elevated risks of other psychiatric illness, analyses of the specificities of these associations have produced mixed findings. Extensive evidence links both past-year and lifetime cannabis use and CUD with other substance use, with the strongest associations for alcohol use compared to other illicit drugs. Although directionality is implied that cannabis could be one potential “gateway” to later use of other substances, the role of cannabis as the causal factor in other illicit drug use remains to be clarified, due to the potential effect of common pre-existing risk factors for both cannabis and other substances. Nonetheless, the clear association of cannabis use with later illicit drug use suggests the need for appropriate intervention strategies for those at risk of developing a CUD, in order to diminish later SUD risk. Furthermore, risk of concurrent CUD and OUD should be considered in clinical contexts. American Medical Association (AMA) guidelines currently state that regular use of cannabinoids should not be a reason to suspend medication use in treatment of addiction involving opioids [146]. At the same time, given that states increasingly permit legal medical and recreational cannabis use, the potential has increased for opioids and cannabinoids to be misused concurrently. Thus, due to the risk for co-occurring CUD with other SUDs, clinicians should carefully evaluate the appropriate psychosocial treatment.

Strong evidence also links cannabis use and psychosis, with additional lines of evidence suggesting that cannabis could potentially be a causal factor in later psychosis. However, the complexities of genetic and additional environmental risk factors complicate this relationship, and the role of cannabis use in cognition of those with schizophrenia has generated inconsistent findings. Thus, further longitudinal research which takes into account confounds will aid in delineating this relationship.

National data suggest that cannabis use and CUD are associated with mood disorders, particularly depression and bipolar I. Twin study models show potential directionality between cannabis use and later depression, however replicated results on larger samples are necessary in order to make firmer conclusions. Furthermore, cannabis use and CUD are higher in those with bipolar disorder than the general population, but little research exists regarding the course of this comorbidity, and inconsistent findings regarding elevated risk factors have been reported [139,140,141]. Much more remains to be clarified about the nature of cannabis use among those with bipolar disorders, and about the impact of such use on the course and prognosis of bipolar disorders. Nonetheless, proportionally higher rates of frequent cannabis use and CUD among those with a mood disorder underscores the clinical importance of cannabis abuse screenings concurrently with mood disorder screenings.

Similarly, CUD is associated with both anxiety disorders and personality disorders, yet evidence regarding the specific role of cannabis in the course of these disorders is limited. More specifically, the role of genetic in risk of CUD among individuals with a personality disorder is complex and not well understood, and more definitive evidence of the relationship of cannabis use with personality disorders could help guide clinical practice. Furthermore, while some data show potential therapeutic benefit of medical cannabis for individuals with PTSD, these results are complicated by a potential increased risk for abuse of cannabis as well as potential long-term harms [41]. Therefore, the increased risk of CUD should be recognized in clinical settings, in conjunction with any potential therapeutic benefits of cannabis for anxiety. The lack of evidence between CUD and co-occurring PTSD is noted in particular, since as of 2020, PTSD is a qualifying condition for medical cannabis approval in 26 states [147]. Thus, additional experimental data and clinical trials investigating the use of cannabis among PTSD patients are needed in order to provide more conclusive evidence of the acute and long-term effects. Similar clinical data are needed for other psychiatric diagnoses qualifying as a condition for medical cannabis approval, for example bipolar disorder, ADHD, and anorexia.

Considering rapidly changing state laws regarding the legality of cannabis, the current disconnect between the cannabis industry, public opinion, and scientific literature is striking. Cannabis is a $13.6 billion industry [148,149,150], with millions spent yearly on lobbying to increase legalization [151,152]. Bearing in mind the potential for poorer prognosis among those with psychiatric comorbidities, if they also use cannabis or have a CUD [36], public and provider education on the current evidence regarding cannabis use, CUD, and a co-occurring disorder is essential to properly guide clinical practice, policy, and decisions of the general public about whether to use cannabis or not.

## 5. Conclusions and Future Directions

Overall, beginning with the Epidemiologic Catchment Area study in the 1980s and accumulating to today, a large body of literature indicates substantial associations between frequent cannabis use, CUD, and additional psychiatric illnesses. Considerable evidence indicates that state medical cannabis laws increase cannabis use and use disorder in adult populations [20,153,154], consistent with evidence indicating that state recreational laws increase adult non-medical cannabis use and CUD [155]. Because of the elevated risk of CUD and co-occurring mental illness, this time of changing cannabis legislation is a critical time to highlight the increased need for effective prevention and treatment strategies for the co-occurrence of CUD with other substance and psychiatric disorders.

One proposed future direction to aid in clarifying the relationship between cannabis use, CUD, and comorbid psychiatric illness is a greater level of standardization of cannabis use definitions in empirical research. Since the frequency of use, potency of cannabis products, and severity of CUD are linked to stronger associations with a comorbid psychiatric diagnosis [28], and cannabis potency is increasing internationally, better measures of the amounts of cannabis consumed could help to elucidate outcomes. Because of this, awareness of THC to CBD ratios as well as amounts of each could help play a role in deciphering mixed findings. However, no standardized, scientifically valid and widely used measure of cannabis exposure exists thus far, a critical gap in the existing body of literature. Creating measures of cannabis exposure faces several challenges, such as a lack of a standard unit to measure cannabis consumption that are analogous to the standard drink units and measures of binge drinking for alcohol consumption. Recent articles have noted the difficulties in measuring cannabis consumption patterns, which are further complicated by inaccuracies in cannabis product labels [156], which have drawn attention to the need for further standardization measures [157] that can be used for clinical as well as research purposes.

The strengths of this narrative review are noted. First, this review aimed to synthesize recent studies regarding cannabis use, CUD, and co-occurring disorders to bring together what is known and identify gaps in the literature in one summation. We believe that by organizing the current literature this way, we are also able to identify points of controversy in the field, for example studies showing opposite results regarding the role that cannabis plays in cognitive function among individuals with schizophrenia [104,105]. This underscores the complex nature of cannabis, and that further research is necessary in order to identify its role for most comorbidities. Limitations of this review are also present. By adopting the narrative review methodology, no quantitative meta-analysis of results or systematic search according to an official guide was conducted, leaving opportunity for this to be done in a future study. Second, the complex dose-dependent effects of cannabinoids on the brain is an additional point to consider when discussing the associations of cannabis use with other psychopathologies. Thus, an updated review of the major compounds found in cannabis and their relationship with co-occurring disorders is also needed.

Finally, given the extent of comorbidity of cannabis use, CUD, and other comorbid substance and psychiatric illnesses, greater guidance is needed for clinicians in terms of education about potential risks, methods of assessment and monitoring, and the most effective treatment strategies among patients with cannabis use or CUD and substance use or psychiatric disorders. For patients with comorbid substance use disorders, clinician guidance is needed to determine which substances merit treatment focus when more than one is involved (the most common case), and how to prioritize that focus when more than one substance is involved. For patients with cannabis use or CUD and psychiatric disorders, guidance may be needed on modifications in behavioral treatments and/or medication strategies.

## Figures and Tables

**Table 1:** Adjusted Odds Ratios (aORs) ^a^ indicating the association of CUD with a psychiatric disorder National Epidemiologic Survey on Alcohol and Related Conditions (NESARC) 2001–2002 and NESARC-III 2012–2013.

	Any 12-Month Cannabis Use Disorder		Any Lifetime Cannabis Use Disorder	
	NESARCaOR (95% CI) ^b^	NESARC-III aOR (95% CI)	NESARCaOR (95% CI)	NESARC-III aOR (95% CI)
Any other substance use disorder	-- ^c^	9.3 (7.70–11.21)	--	14.5 (11.95–17.60)
Alcohol use disorder	7.8 (6.21–9.89)	6.0 (5.10–6.97)	10.3 (9.15–11.66)	7.8 (6.95–8.74)
Any other drug use disorder	--	9.0 (6.65–12.19)	--	10.0 (8.56–11.76)
Nicotine use disorder	--	6.2 (5.24–7.34)	--	6.6 (5.79–7.64)
Nicotine dependence	5.1 (4.19–6.31)	--	5.2 (4.67–5.79)	--
Any mood disorder	2.9 (2.28–3.60)	3.8 (3.10–4.56)	2.9 (2.63–3.26)	3.3 (2.94–3.73)
Major Depressive Disorder	1.8 (1.29–2.52)	2.8 (2.33–3.41)	1.9 (1.69–2.14)	2.6 (2.26–2.95)
Bipolar I	3.1 (1.77–5.48)	5.0 (3.65–6.75)	2.5 (2.08–3.10)	3.8 (3.10–4.59)
Bipolar II	4.3 (3.02–6.14)	2.7 (1.10–6.62)	4.3 (3.59–5.08)	2.8 (1.51–5.23)
Dysthymia	1.9 (1.09–3.28)	--	2.3 (1.71–2.95)	--
Any anxiety disorder	2.4 (1.90–3.15)	2.8 (2.24–3.39)	2.4 (2.19–2.73)	2.9 (2.54–3.31)
Panic Disorder	--	3.3 (2.50–4.48)	--	3.2 (2.66–3.76)
Panic with agoraphobia	4.9 (2.86–8.36)	--	3.9 (2.84–5.34)	--
Panic without agoraphobia	2.6 (1.59–4.09)	--	2.7 (2.23–3.20)	--
Agoraphobia	--	2.6 (1.64–4.06)	--	2.9 (2.25–3.79)
Social Phobia	2.4 (1.61–3.57)	2.3 (1.61–3.27)	2.4 (1.98–2.78)	2.7 (2.22–3.40)
Specific Phobia	2.2 (1.60–3.04)	1.7 (1.28–2.29)	2.2 (1.92–2.52)	2.1 (1.73–2.46)
Generalized Anxiety Disorder	4.3 (2.75–6.70)	3.7 (2.79–5.02)	2.7 (2.25–3.19)	3.2 (2.75–3.74)
Post-Traumatic Stress Disorder	--	4.3 (3.26–5.64)	--	3.8 (3.15-4.67)
Any personality disorder	3.9 (3.18–4.66)	4.8 (3.96–5.75)	3.2 (2.87–3.55)	4.7 (4.18–5.28)
Antisocial	6.0 (4.66–7.79)	3.8 (3.05–4.75)	6.7 (5.70–7.92)	4.7 (4.07–5.34)
Avoidant	2.6 (1.80–3.85)	--	2.7 (2.10–3.37)	--
Dependent	7.2 (3.96–13.07)	--	3.6 (2.24–5.86)	--
Obsessive-Compulsive	2.5 (1.92–3.31)	--	2.1 (1.84–2.42)	--
Paranoid	2.9 (2.13–3.86)	--	2.7 (2.32–3.22)	--
Histrionic	4.1 (2.92–5.84)	--	3.2 (2.59–3.83)	--
Schizoid	2.7 (1.98–3.77)	--	2.5 (2.08–3.07)	--
Schizotypal	--	4.4 (3.60–5.46)	--	4.0 (3.46–4.72)
Borderline	--	5.0 (4.13–6.10)	--	4.5 (3.96–5.19)

^a^ ORs were controlled for sex, race/ethnicity, age, marital status, education, household income, urbanicity, and region at both time points. ^b^ CI: Confidence Interval. ^c^ Due to the use of DSM-IV to measure psychiatric disorders in the NESARC, and DSM-5 to measure the same disorders in NESARC-III, not every diagnosis matched up. Thus, diagnoses not in one paper are indicated by ‘--’.

**Table 2:** DSM-5 Major Depressive Disorder criteria, and overlapping cannabis withdrawal symptoms.

DSM-5 MDD Criteria and Overlapping Symptoms in CWD.	DSM-5 Major Depressive Disorder	DSM-5 Cannabis Withdrawal Syndrome
Depressed mood—indicated by subjective report or observation by others (in children and adolescents, can be irritable mood).	✓	✓
Loss of interest or pleasure in almost all activities—indicated by subjective report or observation by others.	✓	
Significant unintentional weight loss/gain or decrease/increase in appetite	✓	✓
Sleep disturbance (insomnia or hypersomnia).	✓	✓
Psychomotor changes (agitation or retardation) severe enough to be observable by others.	✓	✓
Tiredness, fatigue, or low energy, or decreased efficiency with which routine tasks are completed.	✓	✓
A sense of worthlessness or excessive, inappropriate, or delusional guilt (not merely self-reproach or guilt about being sick).	✓	
Impaired ability to think, concentrate, or make decisions—indicated by subjective report or observation by others.	✓	
Recurrent thoughts of death (not just fear of dying), suicidal ideation, or suicide attempts.	✓	

## Data Availability

Data sharing not applicable.
